# Where have the microbubbles come from ? Left inferior pulmonary arteriovenous fistula detected by transoesophageal echocardiography

**DOI:** 10.1093/ehjimp/qyae052

**Published:** 2024-05-24

**Authors:** Ryo Horita, Daisuke Hachinohe, Yuhei Kasai, Ryo Otake, Hidemasa Shitan, Tsutomu Fujita

**Affiliations:** Department of Cardiovascular Medicine, Sapporo Cardio Vascular Clinic, Sapporo Heart Center, North 49, East 16, 8-1, Higashi Ward, Sapporo 007-0849, Japan; Department of Cardiovascular Medicine, Sapporo Cardio Vascular Clinic, Sapporo Heart Center, North 49, East 16, 8-1, Higashi Ward, Sapporo 007-0849, Japan; Department of Cardiovascular Medicine, Sapporo Cardio Vascular Clinic, Sapporo Heart Center, North 49, East 16, 8-1, Higashi Ward, Sapporo 007-0849, Japan; Department of Cardiovascular Medicine, Sapporo Cardio Vascular Clinic, Sapporo Heart Center, North 49, East 16, 8-1, Higashi Ward, Sapporo 007-0849, Japan; Department of Cardiovascular Medicine, Sapporo Cardio Vascular Clinic, Sapporo Heart Center, North 49, East 16, 8-1, Higashi Ward, Sapporo 007-0849, Japan; Department of Cardiovascular Medicine, Sapporo Cardio Vascular Clinic, Sapporo Heart Center, North 49, East 16, 8-1, Higashi Ward, Sapporo 007-0849, Japan

**Keywords:** pulmonary arteriovenous fistula, echocardiography, microbubble test

A 77-year-old female, suspected of cardiogenic stroke, was referred to our hospital to investigate the embolic source of cerebral infarction (*Figure 1A*). The electrocardiogram showed sinus rhythm with no evidence of atrial fibrillation (AF). Transthoracic echocardiography with a microbubble test showed grade 4 right-to-left shunt (RLS) after four cardiac cycles (*Figure 1B*, Supplementary data online, *Video S1*). Transoesophageal echocardiography (TEE) demonstrated grade 3 RLS; however no microbubbles passed through patent foramen ovale, even with Valsalva manoeuver (*Figure 1C*, Supplementary data online, *Video S2*). TEE also revealed the substantial flow of microbubbles from the left inferior pulmonary vein (LIPV) to the left atrium, suggesting a pulmonary arteriovenous shunt (*Figure 1D*, Supplementary data online, *Video S3*). Contrast-enhanced computed tomography (CT) detected a pulmonary arteriovenous fistula (PAVF) flowing into LIPV at the left lower lobe (*Figure 1E*).

**Figure 1:**
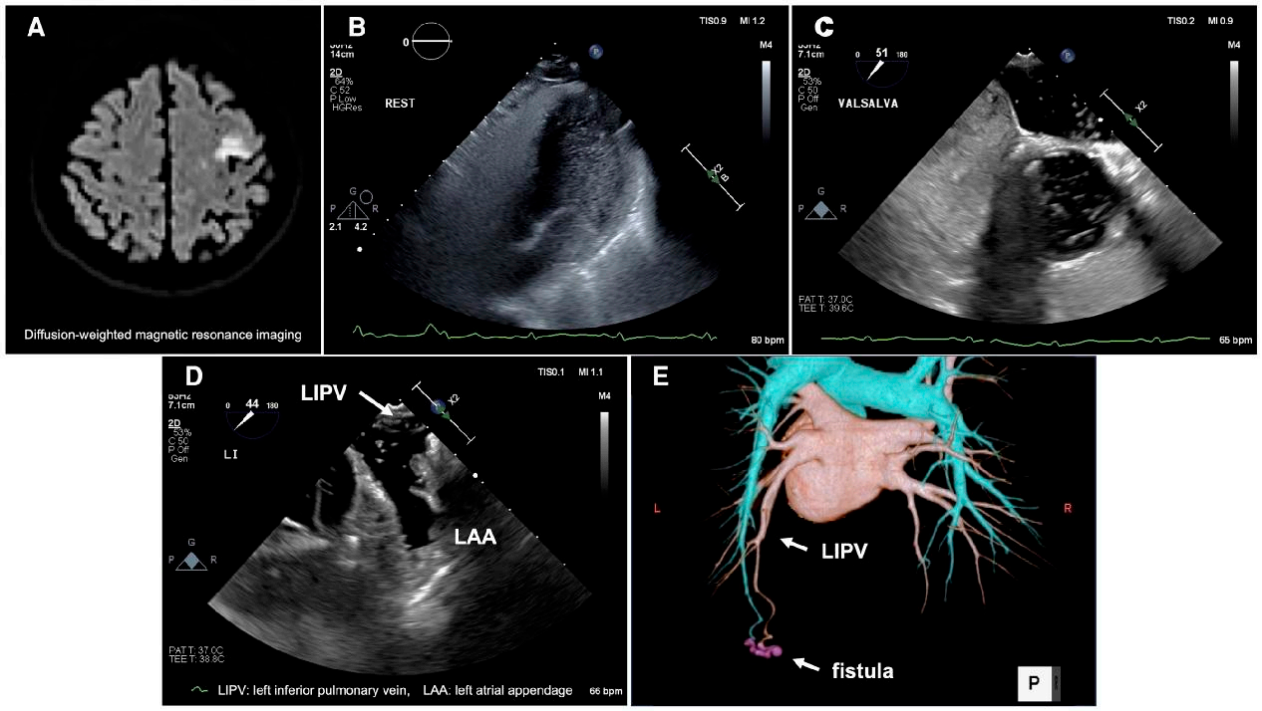
The patient's imaging findings.

The microbubble test effectively screens for paradoxical embolism. In this case, microbubbles flowing from LIPV after four cardiac cycles indicated the presence of PAVF. The gold standard for diagnosis of PAVF is CT, and the combination of echocardiography and CT is effective in detecting PAVF. The various symptoms of PAVF such as dyspnoea, cyanosis, and intrapulmonary haemorrhage give us a chance to suspect stroke caused by PAVF. Transcatheter embolization is performed to prevent recurrence; however, since implantable cardiac monitor recorded AF, anticoagulation therapy was initiated and embolization for PAVF was deferred in this case.

It highlights the importance of thoroughly investigating extracardiac shunts in similar situations.

## Supplementary data

Supplementary data are available at *European Heart Journal – Imaging Methods and Practice* online.

## Consent

The authors confirm that written consent for the submission and publication of this case report, including images and associated text, has been obtained from the patient.

## Supplementary Material

qyae052_Supplementary_Data

## Data Availability

The data underlying this article will be shared on reasonable request to the corresponding author.

## Lead author biography

Dr Ryo Horita graduated from Faculty of Medicine, Wakayama Medical University, in 2014. Currently, he works as a cardiologist at Sapporo Cardio Vascular Clinic. He spends most of his time working on structural heart disease as an interventionist and echocardiologist.

